# Can Grassland Chemical Quality Be Quantified Using Transform Near-Infrared Spectroscopy?

**DOI:** 10.3390/ani12010086

**Published:** 2021-12-31

**Authors:** Silvia Parrini, Nicolina Staglianò, Riccardo Bozzi, Giovanni Argenti

**Affiliations:** Department of Agriculture, Food, Environment and Forestry (DAGRI), University of Florence, 50144 Florence, Italy; nicolina.stagliano@unifi.it (N.S.); riccardo.bozzi@unifi.it (R.B.); giovanni.argenti@unifi.it (G.A.)

**Keywords:** meadows, NIRS, botanical composition, forage quality, quantification

## Abstract

**Simple Summary:**

Near-infrared spectroscopy (NIRS) has been applied to analyse the quality of forage and animal feed. However, grasslands more than other raw materials are linked to many variability factors (e.g., site, year, occurring species, etc.) that can represent strong points as well as weak points in NIRS estimation. This research is aimed at testing NIRS application for the determination of chemical characteristics of fresh, undried and unground samples of meadows and grasslands located in north-central Apennine. The interest lies in the possibility of monitoring grassland resources, supporting the decision in terms of the need of supplementation and identifying the critical periods for cutting grassland intended for animal feeding. The results indicated that FT-NIRS models could be used in the real-time quantification of crude protein, fibrous fraction and dry matter, while for lignin only a screening test could be considered. Minor components of grassland such as ash and lipids need improvement. As a practical point, a key factor of FT-NIRS in grassland chemical quality estimation is the absence of samples preparation and the importance of the parameters that have obtained the best results in animal diet formulation.

**Abstract:**

Near-infrared spectroscopy (NIRS) and closed spectroscopy methods have been applied to analyse the quality of forage and animal feed. However, grasslands are linked to variability factors (e.g., site, year, occurring species, etc.) which restrict the prediction capacity of the NIRS. The aim of this study is to test the Fourier transform NIRS application in order to determine the chemical characteristics of fresh, undried and unground samples of grassland located in north-central Apennine. The results indicated the success of FT-NIRS models for dry matter (DM), crude protein (CP), acid detergent fibre (ADF), neutral detergent fibre (NDF) and acid detergent lignin (ADL) on fresh grassland samples (R2 > 0.90, in validation). The model can be used to quantitatively determine CP and ADF (residual prediction deviation-RPD > 3 and range error ratio- RER > 10), followed by DM and NDF that maintain a RER > 10, and are sufficient for screening for the lignin fraction (RPD = 2.4 and RER = 8.8). On the contrary, models for both lipid and ash seem not to be usable at a practical level. The success of FT-NIRS quantification for the main chemical parameters is promising from the practical point of view considering both the absence of samples preparation and the importance of these parameters for diet formulation.

## 1. Introduction

The importance of maintaining grassland systems is now well known because failure to maintain these systems is linked to the loss of biodiversity [1] and the reduction of ecosystem stability [2]. Land use intensification and homogenization of landscapes [3,4], as well as the lack of agriculture adaptability to changing environmental conditions, could affect the maintenance of ecosystems and the services they provide [5]. The main service supplied by grassland ecosystems is the supplying of forage requirements for ruminants both in terms of quantity and quality [6,7]. The decision on integration needs is essentially based on the quantity and quality assessments of the grassland. The first, easy to assess, is determined by the yield, while the second represents the nutritional value available for animals, which is more difficult to evaluate in terms of both sampling and analysis.

A further concern is the continuous variation of grassland quality, linked to numerous direct factors such as species composition and variety [8], abundance [9], phenological phase and growing condition [10], soil resource availability [11] and management practices [12]. In the long term, indirect factors can affect the variability of grasslands [13]; in this context climate change can impact crop and forage resources, with effects on the growing season length and the ripening of species, but also on the yield and species distribution [14].

Since the first application in 1976, feed evaluation research has been evolving to replace traditional wet chemistry analyses with near infrared spectroscopy (NIRS). This technique can be successfully applied in grassland systems because plants consist of structural and soluble carbohydrates, protein, fat, and organic acids that chemically contain the most relevant groups (C–H, N–H, O–H) for potential NIRS identification [15]. NIRS is based on the absorption of photon energy and the excitation of molecular overtones and combined vibrations from chemical groups containing mainly hydrogen [16].

In natural science, NIRS and other spectroscopy methods, such as Fourier transform near-infrared spectroscopy (FT-NIRS) and visible near-infrared spectroscopy (VISNIRS), have been implemented in basic and applied science [17], including in studies of animal feed composition. It is known that NIRS has the capacity to estimate chemical compositions of several feeds, including dried mixed forages and silages [18,19], *Medicago sativa* [20,21], *Zea mays* [22] as well as whole cereal plants [23] and woody forage [24]. With regard to natural grasslands, the vast majority of studies reported success for the estimation of chemical composition of dried ground samples [25,26,27,28]. Other works [29,30] described the application of NIRS to fresh samples of herbage in order to reduce the time of analysis, considering that the high water content affects the spectra results. The effect of samples preparation has been considered by Alomar et al. [29], who reported the fresh herbage of Southern Chile pasture, while Reddersen et al. [30] studied the effect of sampling conditions (standing sward, silage, hay/chopping and milling) on fresh grassland biomass. In vegetation ecology, NIRS has been implemented for discrimination analysis of functional types and single species [31].

Considering the spatial and temporal variation of meadows and pastures in terms of botanical composition, the high cost of traditional analyses and the long waiting times, NIRS gives the opportunity for fast and efficient analysis of large numbers of samples. In this context, the application of the Fourier transform algorithm to NIRS (FT-NIRS) could allow for both an improved spectral resolution and a reduced scan time. However, NIRS always needs to be calibrated, and the application of a multivariate model is always necessary in order to compare spectral results with the samples with known compositions. Partial least square regression (PLS), as well as other mathematical approaches (principal component regression (PCR)) or techniques of statistical learning (artificial neural networks, Random Forest) were applied for the regression model developments. For the model validation, cross validation or an internal test set of samples were often used, even if those models’ systems may cause fewer errors with respect to the use of external data sets of validation [32]. Karayilanli [33] reported that validation models demonstrate great accuracy in closed populations, referring to a subset of the validation samples included in calibration data sets or belonging to the same natural population, and that forage crops could be represented by the same harvest or field [32]. NIRS estimation capacity should not decrease the accuracy if a sample from other harvests or of different origin is predicted. Indeed, a less common adjustment was necessary, using a more variable initial calibration set, while validation was often much less successful.

The validation of data including many variables, such as data from pastures and meadows, and influenced by numerous variability factors, should be solved in order to practically apply the NIRS approach. In fact, considering the global change scenarios, there is a need of increasing research and monitoring of forage quality of grassland systems [7]. However, nutritional quality monitoring is rarely done due to the complexity of determining forage quality, the high variability of natural systems and low financial interest.

The aim of this study is to test the NIRS application in order to determine the chemical characteristics of fresh, undried and unground samples of meadows and grasslands located in north-central Apennine. At the practical level, the interest lies in the possibility of monitoring grassland resources, supporting the decision in terms of the need of supplementation and identifying the critical periods for cutting grassland intended for animal feeding. 

## 2. Materials and Methods

### 2.1. Forage Sample Set 

The study used 150 samples collected in the period of 2014–2019 in seminatural and artificial grasslands in north-central Apennine at an altitude between 300 and 1174 m.a.s.l.

The experimental sites for each area were chosen for the representativeness of the respective territory. Each site was sampled at least two times during the growing season, including both primary growth and secondary regrowth. Samples were obtained from an area of 1 sqm. The area of study included meadows intended for cutting and pastures dedicated to animal grazing. Samples were obtained from sites that encompassed 4 groups:

i. Old alfalfa meadows re-colonized by spontaneous species (*n* = 35);
ii. Grass–legume mixtures recently established (*n* = 30);
iii. Old legume mixtures grassland re-colonized by native species (*n* = 60);
iv. Alfalfa crops recently established (*n* = 25).

The botanical composition was assessed inside the same sample plots for each cutting. The percentage proportion of each botanical species was estimated visually as the percentage contribution to the herbage mass [10]. Herbaceous species were grouped in grasses, legumes and other forbs, as is usually performed in forage research [34].

Old alfalfa pure stands(i) (8–12 years old) were highly naturalized by local species belonging to grasses and forbs, and the ground cover of alfalfa was reduced, even if this percentage became higher in the second and third cut.

Recent grassland mixtures(ii) (less than 4 years of age) presented a high presence of sown species (about 70–80% as the annual average), mainly represented by *Medicago sativa* or *Trifolium* sp., *Dactylis glomerata* being the most frequently occurring grass. 

Old legume mixtures grassland re-colonized by native species(iii) presented about 65% of grass species (belonging to genus *Dactylis*, *Festuca*, *Lolium*, *Avena*, *Poa* and *Bromus*) and 25% of legumes and the rest of the other forbs.

Recent alfalfa crops(vi) (2–4 years old) presented a percentage of the sown legume around 75–85%, with the main weeds represented by grasses (belonging to genus *Lolium*, *Poa* and *Bromus*).

### 2.2. Sample Preparation and Spectral Measurement

Fresh herbage samples (stalks and leaves) were cut by hand shears to 2–4 cm in length and mixed by hand. For each sample, three sub-samples weighing about 30 g were randomly selected. After this procedure, while maintaining a constant temperature, each sub-sample was exposed by a cup spinner to an electromagnetic scan over the wavenumber range of 4000–9999 cm^−1^ and corrected against the background spectrum. For each aliquot, a spectral measurement was obtained from 32 scans performed using an FT-NIRS Antaris II model (Thermo Scientific, Waltham, MA, USA) at a lab room temperature of approximately 20–21 °C. Spectral data were collected as reflectance (R) and converted as absorbance (log1/R). The average spectrum of the three measurements was calculated for each sub-sample and used as the final spectrum.

### 2.3. Chemical Analysis

After the FT-NIRS collection, the samples were dried in a forced-air oven at 60 °C at constant weight and grounded through a mill (Brabender OHG, Duisburg, Germany) to pass 1 mm. The main chemical components were determined according to the AOAC [35] methods: dry matter (DM) content using the 934.01 method, crude protein (CP) by the 976.05 method, ash via the 942.05 procedure, crude fat (CF) using the 2003.05 method, acid detergent fibre inclusive of residual ash (ADF) and acid detergent lignin (ADL) using the 973.18 method. Neutral detergent fibre, inclusive of residual ash (NDF), was analysed in accordance with Van Soest et al. [36].

### 2.4. Statistical Analysis

For the model development, we randomly split the available samples into a calibration set (80%) and a validation set (20%) within each group (i, ii, iii and iv), ensuring that samples of similar botanical composition were present in both data sets. 

For each chemical constituent (DM, CP, ash, CF, NDF, ADF and ADL), an individual model was developed and the entire measured spectra region (3999–9990 cm^−1^) was considered. A single spectral pre-treatment option, or a combination of options, was applied to spectral data prior to calibration of the model. 

First derivate of the spectral data was calculated in order to correct the light scattering contributions [37]; however, it did not correct the pathlength variation, for which multiplicative scatter correction or standard vector normalization were necessary. 

The Savitzky–Golay smoothing filter was useful for improving the appearance of peaks obscured by random noise, considering 3 data points and a polynomial order. By contrast, in some cases smoothing was not necessary. 

Mathematical pre-treatment was specific for each parameter and remained identical both in calibration and in validation. 

Processing of spectral data was performed to identify outliers with the TQ Analyst software [38], guaranteeing the removal of outliers below 2% of the population, as suggested by Williams et al. [39].

Lastly, PLS was applied to predict the chemical composition of samples, setting the upper limit at 10 PLS factors. A good model, in fact, should have as few independent latent variables or principal components as possible. The optimal number of PLS factors used for model development was that which determined the lowest error in cross-validation, also considering the results of the PRESS (predicted residual error sum square).

The quality of NIRS calibrations was evaluated in terms of the highest coefficient of determination (R^2^), which represents the proportion of the explained variance of the response variable in the calibration (R^2^) or validation dataset (R^2^v). Errors were evaluated in terms of the lower root mean square error in calibration (RMSEC) and in validation (RMSEV). Small differences between RMSEC and RMSEV were always preferred.

The residual prediction deviation (RPD), the ratio between the standard deviation (SD) of the reference values and the mean error of prediction, was calculated as the qualitative assessment of the results. A small error of prediction compared to reference values meant a high RPD value resulting in a good model. Pérez-Marín et al. [40] reported that when reference data variance is low, the values for the R^2^ and the RPD cannot be very high.

The model can be considered sufficient for a screening if RPD is between 1.5 and 2.5 [41]. Some authors, i.e., Williams and Sobering [42], suggested an accurate estimation capacity if the RPD values were higher than the limit of 2.5, even though in the following years it seems that the limit of accuracy evaluation was increased to 3 [41]. However, higher values for the RPD suggest increasingly accurate models.

Furthermore, the relationship between the range of composition of the reference data and the RMSEP, known as the range error ratio (RER) index [40], was calculated. The RER was considered as statistical indicators with the greatest weight in the precision of an NIRS calibration model [43]. The RER values in the range of 4–8 suggest the possibility of discriminating between high and low values, while RER values in the range of 8–12 represent the possibility of predicting quantitative data [44,45].

## 3. Results

### 3.1. Near-Infrared Spectra 

**Figure 1:**
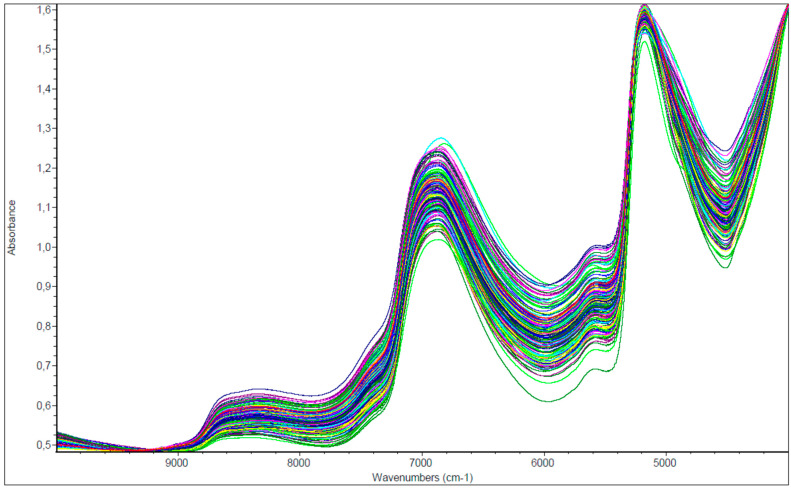
Original NIR spectra (*n* = 150) of entire data set: each colour represents one sample spectrum in the near-infrared region.

Original near-infrared spectra (Figure 1) show high peak regions around wavenumber between 6800 and 6900 cm^−1^, 5100–5200 cm^−1^ and a slope at 5600 cm^−1^.

### 3.2. Descriptive Statistics 

The mean, standard deviation, median and range of the chemical composition of the data set obtained by wet chemistry are shown in Table 1. The samples are reported after the removal of outliers and split into calibration and validation sets. The results of traditional chemical analyses show a wide range of values for most of the parameters, except for crude fat, which presented the narrowest range. This result was expected and approved due to the different origins of the samples: years, sites, origins, botanical composition and phenological phases.

Both in calibration and in validation, the descriptive statistics of the parameters showed the same behaviour. Mean and median were similar in all parameters even if Crude protein, ADF and ADL had higher median values compared to mean values, indicating that a proportion of the population had a higher value than the rest of the population. By contrast, the distribution of reference data seems to be within the sample population; in fact, dry matter and NDF each had a lower median than mean, as well as a high SD, suggesting that a proportion of the population was lower than the rest according to the considered parameters. Ash and crude fat each presented a similar mean and median, and were also associated with the lowest SD.

### 3.3. NIRS Models

A summary of the statistics for the performance of the calibration and validation models are reported in Table 2. For each parameter, the optimal number of PLS factors used, as well as the mathematical treatments, are included. The NIRS regions used for each chemical constituent are also specified. For dry matter the area referred to a combination of O–H stretching, including from 4800 to 7100 cm^−1^, was used. The optimal wavenumber for CP was both 4800–5200 cm^−1^ (1923–2080 nm) and between 6200 and 7200 cm^−1^ (1389–1613 nm), which corresponded to the combination of N–H stretching. For ash the full available near-infrared region was used, while for crude fat only the initial and final part of the NIRS region (5100–9200 cm^−1^) were excluded. For the NDF, the region considered, from 5500–6200 cm^−1^ (1612–1818 nm), was the same as that for ADF, while ADL was referred to a larger area, including in 5183–8333 cm^−1^ (1200–1930 nm).

The MSC or SNV mathematical pre-treatment appeared always to be necessary, aswas true of the first or second derivative. By contrast, smoothing was not essential and was applied only in some cases. 

The result of the calibration of the NIRS models (Table 2) reported coefficients of determination higher than 0.90 for DM, CP and all fibrous fractions (NDF, ADF, ADL). Nevertheless, in external validation R^2^ values were from 1% to 1.5% lower for DM, CP and ADF, while they were slightly higher for NDF and ADL (5% and 3% higher in validation than in calibration, respectively). Both ash and crude fat obtained a medium R^2^ in calibration, in particular for the lipid parameter, but in validation this coefficient became lower. The figures of the models obtained by the main components—DM, CP, NDF, ADF and ADL—are reported as supplementary material (Figures S1–S5, respectively).

Root mean square errors were similar in calibration and validation, even if ash and crude fat showed, also in this case, the worst behaviour in external validation, with higher errors. The RPD results were above 3 for CP and ADF, although values close to this limit were obtained for NDF and DM (2.9 and 2.7, respectively). Encouraging was the RPD (2.3) obtained from ADL, which is sufficient for screening, while ash and crude fat were below the 1.5 limit and need improvement.

For all parameters, the RER numbers were around four to five times larger than those for the RPD. The RER were higher than 8 for DM, CP, NDF, ADF and ADL, suggesting the success in the quantitative prediction for those constituents, while the ~5.4 value of ash and CF might suggest the possibility of discriminating between high and low values.

## 4. Discussion

The resulting descriptive statistics were straightforwardly linked both to different the phenological phase of the samples, and consequently tissue aging development, and to the different forage resources considered. On the other hand, the wide range of the chemical composition can also suggest that the samples are representative of the inherent variability of different fields, sites and years of sampling. The high water content of fresh herbaceous samples (~80%) determined the dominance of the water absorption features (bands) in the NIR spectrum, as reported for other biological products, such as fruit, by Magwaza et al. [46].

Grassland spectra showed broad and strong water-absorption features at about 6944 and 5155 cm^–1^ (1440 and 1940 nm), which were characteristics of the vegetation region related to moisture content in the biological samples [47]. 

The optimal wavenumber model for CP corresponded both to the combination of N–H stretching and to the first overtone of N–H stretching, as suggested by Stuart [48]. Ash cannot be associated with an NIRS region probably due to the absence of energy absorption of inorganic substances as minerals. Regarding crude fat, the selection of the region should be possible due to the characteristic aliphatic –CH adsorption [48]. Nevertheless, the accuracy of models was not enhanced if the specific region was selected for lipids, probably due to low tissue concentration [16]. Consequently, it seems that only the NIRS region that excluded spectra information might be avoided (5100–9200 cm^−1^). Contrarily, Berauer et al. [7], working on the extraction of information from the spectra, built models using only 1.3% and 7.7% of the spectra wavelengths, respectively, for ash and fat. 

According to Lugassi et al. [49], a specific absorption was shown at 5500 and 5700 cm^−1^ (~1700 nm) that could be linked to organic bonds of plant biochemicals due to the presence of lignin and cellulose [50]. Schwanninger et al. [51] reported that wavelengths around 1715 nm and 1735 nm result from overtone C–H stretching vibrations in polyoses (hemicellulose) and cellulose. A larger area seems necessary for the prediction of lignin according to Li et al. [52], who reported the wavelength at 1243 nm related to the first overtone of phenolic O–H stretching in lignin [53,54] and identified a larger set of relevant wavelengths between 1450 nm and 1700 nm.

Nevertheless, the detection of effective absorption bands was relatively wide and complex in hydrated objects because they were characterized by complex hydrogen bonding interactions between water, sugar, protein, etc. Wavelength drift or shift in informative peaks may be due to differences in chemical composition, in the temperature or in the structure of the samples that cause variations in the optical path [55]. Slight differences in the physical structure of samples can affect the penetration of light, which resulted in high absorbance in our samples. Moreover, Cougnon et al. [56] reported that environmental variation and small differences in the preparation of samples can also affect the resulting equations. 

The selection of wavebands based on known chemical functional groups [30], as performed in this study, in order to by-pass the large water absorption band of fresh samples, allows on the one hand for work on the reduced data set, but on the other may reduce the available information of the full NIRS spectra. On the contrary, Biewer et al. [57] obtained better results with the full spectrum for legume–grass swards instead of selecting wavebands.

As regard the mathematical pre-treatments on spectra, according to Elle et al. [58] standard normal variate (SNV) and multiplicative scatter correction (MSC), specifically intended to corrected NIRS spectra noise, seem to be always necessary to delete the scatter radiation [59]. In addition, our results showed that first-or-second-derivative standardization as well as smoothing are also necessary to enhance the accuracy of unprocessed spectra (raw spectra), as reported in previous studies [60]. Savitzky–Golay filter smoothing could improve the contributions of part of the spectral signal distortion in the data. Nevertheless, it remains difficult to select a priori the best pre-processing method, and the aims of the spectral pre-treatment are mainly to avoid the use of incorrect pre-processing and to enhance the accuracy of the spectra [61].

The better models in terms of R^2^ in validation were obtained for DM, ADF, CP and NDF, while slightly lower results were shown by the lignin component. The RPD and RER indexes, considering additional criteria for determining the prediction utility of each, showed an absence of an exact relationship between those values, probably due to the distribution of samples in the test validation set. However, the results seem to be applicable for all those parameters, even if ADL parameters are in need of improvement and might be used only for the screening of high and low values (based on RPD and RER values).

Several studies on roughage animal feed suggested the better regression models obtained for DM, CP and NDF [27,28,30], while ADF, not considered in all the research, produced contradictory results [26]. However, most research focused on dried and milled samples, while the use of fresh samples can mainly interfere with the DM prediction. It is well known that differences in the physical structure of particle size, moisture content, as well as other biochemical compounds affect spectral the spectroscopic information obtained from the NIR measurement [59] even if spectra pre-treatment has been applied.

A good result, however, was obtained for protein thanks to the spectral importance of nitrogen associated with the adsorption NH bond [16], which is a major component of proteins and of the larger range of proteins in our samples in comparison to other parameters such as ash or fat.

Regarding the ADF and NDF considered as two important limiting factors for the estimation of the nutritive qualities of feed and forage, contradictory results have been reported. Yang et al. [62], studying dried samples of *Lolium multiflorum* both for NDF and ADF, reported better R^2^ and RPD compared to our study. Berauer et al. [7], working on crude fibre content (that is, the parameter most similar to ADF) by Vis-NIRS measuring did not report suitable results in species-rich mountain pastures. Conversely, according to our study, Chen et al. [63] reported that the NIRS model for NDF was less accurate than those for ADF. 

The ADL models seem to be encouraging especially if compared to past studies on dried samples of mixed pasture by Andrés et al., Danieli et al. and Fekadu et al. [25,26,64], who reported lower results for this constituent. In the main case, the unsatisfactory results were associated with the negative influence of chemical methods used as the reference methods. In our case, the ADL estimation was low in terms of accuracy, causing RPD and RER to be slightly lower than other parameters. Improvements of this model might include artificial sampling, which involves increasing the samples in the specific range of the data set where low numbers of data are present which, in this case, coincide with the tail from 10 to the maximum (Figure S2).

Crude fat, often neglected from a nutritional point of view in forage and plants due to its low content, obtained the lowest results in terms of R^2^, as well as low RPD and RER, suggesting the non-applicability of the model in practical terms. Berauer et al. [7], studying dried and milled pasture samples and analysing fat, reported both slightly higher R^2^ (0.83, 0.73 in calibration and validation, respectively) and RPD (1.69) than our study, yet they were evaluated as sufficient. Even if inorganic substances did not adsorb energy in the near region and consequently did not give spectral information, ash reached good results in terms of R^2^ and RMSE according to Berauer et al. [7]. Nevertheless, the RPD and RER values indicated that the model is not usable.

The models developed seem to be applicable for the estimation of the main chemical characteristics of the grassland components intended for animal feed. The success of the calibrations could be largely attributed to the robust set of environments used for sample collection compared to those used by Karayilanli et al. [33], who only considered alfalfa–grass mixtures. 

Norman et al. [65], assessing the nutritional value of dried forage species in Australia, suggested the importance of including all sources of variability in the calibration set for the following quantification of unknown samples. Nevertheless, those authors reported that when studies considered global calibration, including samples across seasons and sites, they did not perform as well as the calibration based on a unique site and year. 

The results of our research were certainly affected by numerous variability factors, which on the one hand have a negative effect on the accuracy and a large influence on the resulting equations. On the other hand, the high variability of sample conditions (years, sites, species) in both calibration and validation sets allows for a reasonable confidence that the equations will adequately predict the main chemical components of multiple grass species according to Karayilanli et al. [33]. 

The strategy to improve the results of a very different dataset, such as the grassland of our study referring to a very large area, years and different species, could introduce both an increase of the number of samples and the selection of an artificial calibration set. This could be achieved through the addition of a specific data set including known samples and, in particular, a specific set of samples in different proportions where data are scarce.

## 5. Conclusions

The results referring to fresh samples without milling procedure indicated that the FT-NIRS models developed can potentially be used to quantitatively determine both crude protein and ADF, and that suitable models can be achieved to measure DM and NDF; for the lignin fraction, only a screening seemed to be achieved.

The FT-NIRS seems to be able to quantify the main chemical compositions of grasslands deriving from different sites and years as well as with different occurring species. In order to enhance the NIRS quantification capacity of other chemical parameters, improvements could be considered, such as an increase of the number of samples in the calibration regression or the addition of an artificial data set. 

The importance of a “real time” and high-throughput approach, such as NIRS, for estimating the principal parameters of chemical composition relates to the low feed cost and the very short time of analysis, considering that the samples do not have to be dried and milled. From the practical point of view, the successful quantification of the main nutritional component seems promising, because those parameters are the most important features for assuring an optimal diet formulation and, indirectly, both for maintaining high levels of animals’ welfare and for modulating animal greenhouse gas emissions.

## Figures and Tables

**Table 1:** Descriptive statistics of wet chemistry reference analysis of the samples.

Parameters	Calibration						Validation					
	*n*	Mean	Median	SD	min	max	*n*	Mean	Median	SD	min	max
Dry matter g/100 g	115	20.04	18.50	6.71	11.14	43.12	30	20.29	18.87	5.25	12.22	32.00
Crude protein	118	17.73	18.25	4.74	7.43	25.79	30	17.69	18.64	4.54	10.04	25.37
Ash	114	10.52	10.61	2.05	4.49	15.33	30	10.86	10.96	1.23	7.70	12.71
Crude fat	115	2.28	2.32	0.40	1.25	3.09	30	2.35	2.34	0.34	1.49	2.87
NDF	116	51.11	50.61	8.59	32.77	71.68	30	48.53	48.08	7.07	40.04	65.83
ADF	117	34.85	34.96	6.94	22.13	45.49	30	35.61	36.06	4.94	25.00	43.00
ADL	116	6.65	6.94	2.40	1.72	12.36	30	7.27	7.55	1.39	4.00	9.25

Data are expressed as g/100 g DM unless specified; NDF = neutral detergent fibre; ADF = acid detergent fibre; ADL = acid detergent lignin.

**Table 2:** Near-infrared spectroscopy (NIRS) predictive equations (calibration and external validation).

Parameters	FPLS	Range WN (cm^−1^)	Math Treat.	Calibration		Validation		RPD	RER
				R^2^	RMSEC	R^2^v	RMSEv		
Dry matter g/100 g	6	5118–6817 4800–7100	1; 3; 5	0.951	1.88	0.938	1.97	2.7	10.1
Crude protein	6	4800–5200 6200–7200	2; 3; 5	0.905	1.45	0.901	1.48	3.1	10.4
Ash	3	4000–9000	2; 4; 5	0.837	0.73	0.754	1.01	1.2	5.0
Crude fat	3	5100–9200	1; 3; 5	0.737	0.66	0.652	0.24	1.4	5.8
NDF	5	5500–6200	2; 3; 6	0.911	2.45	0.885	2.47	2.9	10.4
ADF	5	5500–6200	1; 4; 6	0.946	1.06	0.936	1.23	4.0	14.6
ADL	10	5183–8333	1; 4; 6	0.908	0.63	0.880	0.60	2.3	8.8

Data are expressed as g/100 g DM unless specified; NDF = neutral detergent fibre; ADF = acid detergent fibre; ADL = acid detergent lignin. FPLS = number of factors in PLS; Range WN = range of wavenumbers; Math Treat. = mathematical pre-treatment; 1 = MSC; 2: SNV; 3 = first derivate; 4 = second derivate; 5 = Savitzky–Golay Filter (data points: 3, polynomial order: 7), 6 = no smoothing. R^2^ = coefficient of determination in calibration; RMSEC = root mean square error of calibration; R^2^v = coefficient of determination in validation; RMSEv root mean square error of validation; RPD = residual prediction deviation in validation; RER = range error ratio in validation.

## Data Availability

Not applicable.

## Supplementary Materials

The following are available online at https://www.mdpi.com/article/10.3390/ani12010086/s1, Figure S1: Regression models of DM, Figure S2: Regression models of CP, Figure S3: Regression models of NDF, Figure S4: Regression models of ADF, Figure S5: Regression models of ADL.
