# Supplementary material for: Can Grassland Chemical Quality Be Quantified Using Transform Near-Infrared Spectroscopy?
Source: Animals (Basel). 2021 Dec 31;12(1):86. doi: 10.3390/ani12010086 (PMC8749596; doi:10.3390/ani12010086)

Supplementary Material

Figure S1. Regression models of DM

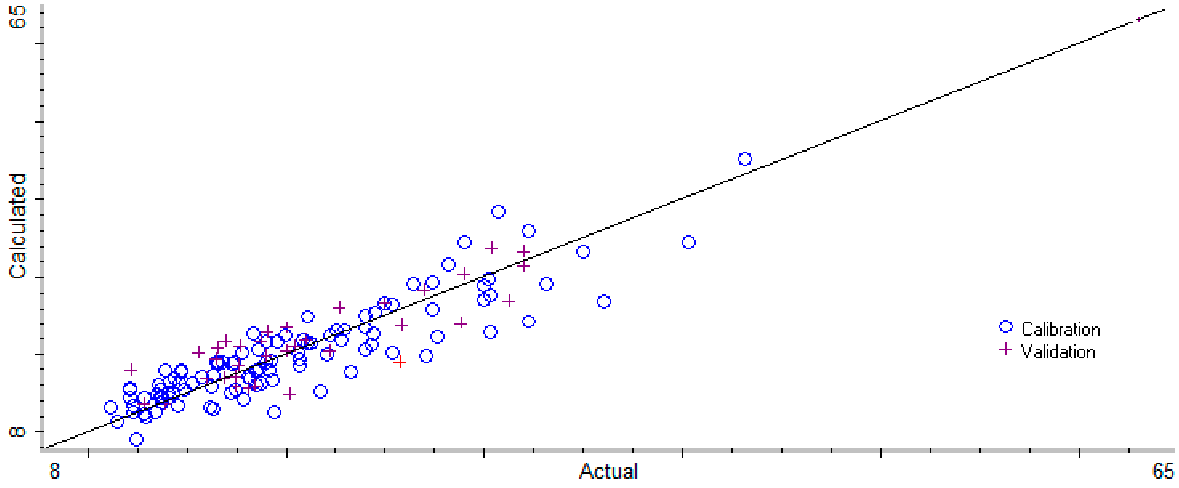

Supplementary Material

Figure S2. Regression models of CP

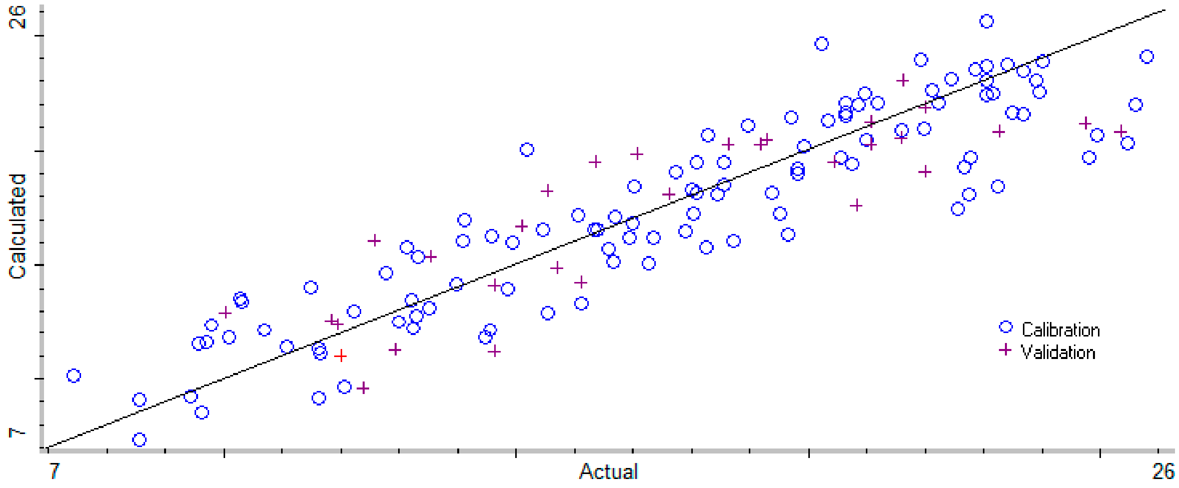

Supplementary Material

Figure S3. Regression models of NDF

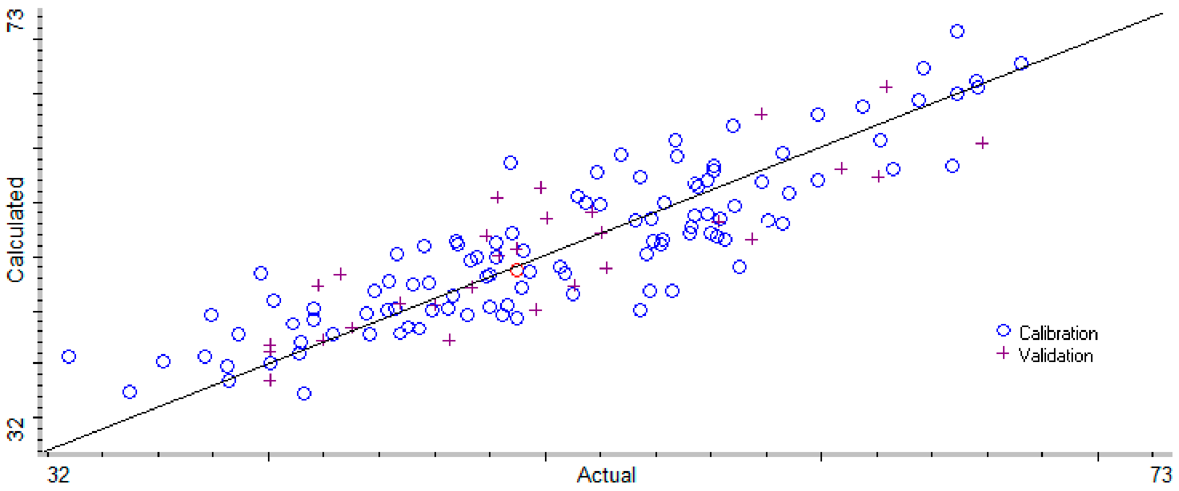

Supplementary Material

Figure S4. Regression models of ADF

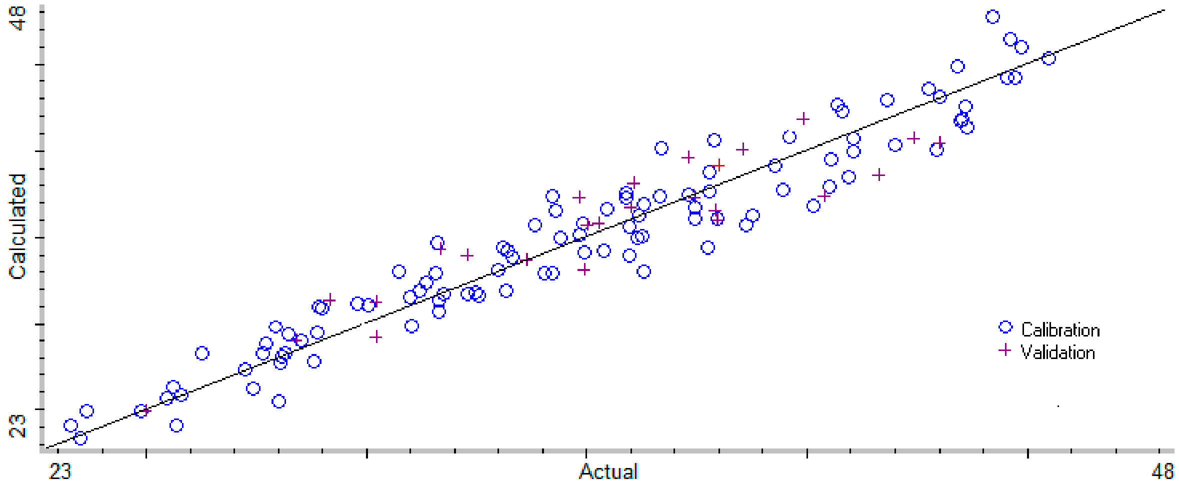

Supplementary Material

Figure S5. Regression models of ADL

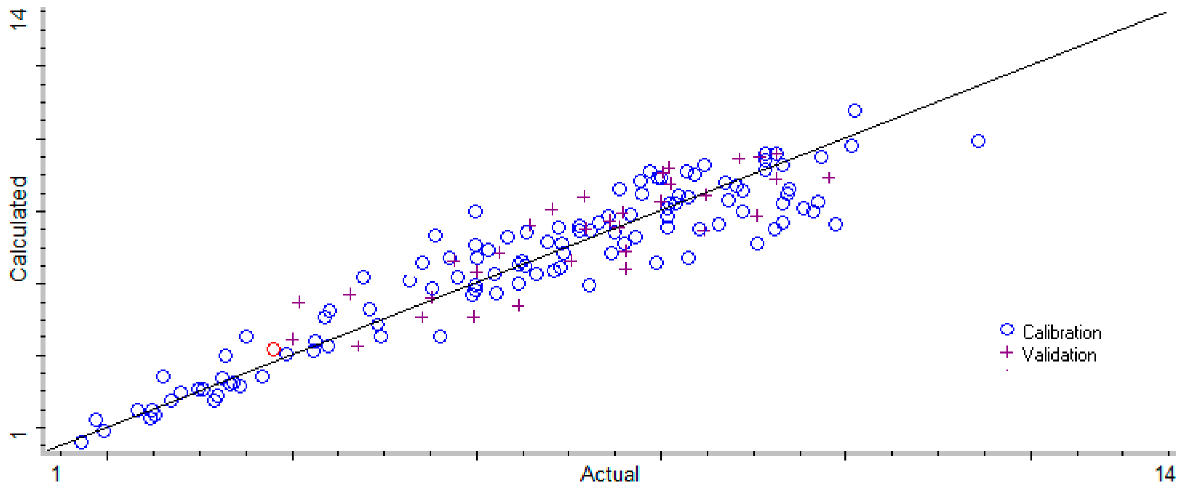

Supplement: Supplementary file 1 [file animals-12-00086-s001.zip › animals-1478591-supplementary.pdf]
